# Krox20 Regulates Endothelial Nitric Oxide Signaling in Aortic Valve Development and Disease

**DOI:** 10.3390/jcdd6040039

**Published:** 2019-11-02

**Authors:** Gaëlle Odelin, Emilie Faure, Corinne Maurel-Zaffran, Stéphane Zaffran

**Affiliations:** 1 Aix Marseille University, INSERM, Marseille Medical Genetics, U1251, 13005 Marseille, France; gaelle.odelin@univ-amu.fr (G.O.); emilie.faure@univ-amu.fr (E.F.); 2 Aix Marseille University, CNRS UMR7288, IBDM, 13009 Marseille, France; corinne.maurel-zaffran@univ-amu.fr

**Keywords:** cardiac development, *Krox20*, nitric oxide synthase, heart, mouse, bicuspid aortic valve

## Abstract

Among the aortic valve diseases, the bicuspid aortic valve (BAV) occurs when the aortic valve has two leaflets (cusps), rather than three, and represents the most common form of congenital cardiac malformation, affecting 1–2% of the population. Despite recent advances, the etiology of BAV is poorly understood. We have recently shown that *Krox20* is expressed in endothelial and cardiac neural crest derivatives that normally contribute to aortic valve development and that lack of *Krox20* in these cells leads to aortic valve defects including partially penetrant BAV formation. Dysregulated expression of endothelial nitric oxide synthase (Nos3) is associated with BAV. To investigate the relationship between *Krox20* and *Nos3* during aortic valve development, we performed inter-genetic cross. While single heterozygous mice had normal valve formation, the compound *Krox20+/−*;*Nos3+/−* mice had BAV malformations displaying an in vivo genetic interaction between these genes for normal valve morphogenesis. Moreover, in vivo and in vitro experiments demonstrate that Krox20 directly binds to *Nos3* proximal promoter to activate its expression. Our data suggests that Krox20 is a regulator of nitric oxide in endothelial-derived cells in the development of the aortic valve and concludes on the interaction of *Krox20* and *Nos3* in BAV formation.

## 1. Introduction

The bicuspid aortic valve (BAV) is a congenital defect found in 1–2% of the population and is the most common valve malformation [1]. A bicuspid valve is comprised of two rather than three semilunar leaflets (or cusps). In humans, most BAVs result from fusion of either the right-coronary and left-coronary leaflets (R-L) or the right-coronary and non-coronary leaflets (R-N) [2]. BAV formation is considered to be an abnormal fusion of the aortic valve leaflets occurring during development [3,4]. Early valve development is a complex process involving the interplay of multiple cell lineages. In the mouse, valve morphogenesis occurs around the embryonic day (E) 9.5 (3 weeks gestation in human) with the formation of endocardial cushions in the outflow tract (OFT) and atrio-ventricular canal (AVC) regions. Development of endocardial cushions initiates with expansion of the cardiac jelly, the extracellular matrix (ECM) between the myocardium and endocardium, followed by endothelial-to-mesenchymal transformation (EndMT) of endocardial cells [5,6]. In the OFT, part of mesenchymal cells colonizing the cushions derive from the neural crest [7,8]. Thus, both endocardial and neural crest derivatives contribute to the development of the aortic and pulmonary valves. At birth, valves continue to develop through apoptosis and remodeling of the ECM [9]. The actual processes that lead to abnormal valvulogenesis and the formation of a BAV are still unclear. However, recent studies have proposed that ECM is crucial for normal development of the aortic valve leaflets [10,11,12]. Neural crest populating the OFT cushions are important for ECM production and late remodeling [13]. We have recently identified a sub-population of the neural crest that contribute to arterial valve development [4]. Neural crest cells are essential for positioning the OFT cushions and patterning the arterial valve leaflets [14]. It is also possible that the valvular endothelium is responsible for transducing luminal events, such as hemodynamic shear stress and generating signals that regulates the developmental program of valvulogenesis. Expression and activity of endothelial nitric oxide synthase (Nos3) in aortic endothelial cells are controlled by hemodynamic shear stress [15]. Endothelial Nos3 plays an important role in aortic valve development, as shown by the presence in *Nos3−/−* mice of partially penetrant R-N BAVs [16] and a study has presented a significant decrease in Nos3 protein amount in BAV compared to tricuspid aortic valve (TAV) human tissues [17]. More recently, second heart field cells have been proposed to contribute to intercalated cushion formation from which the non-coronary leaflet arises [18,19,20]. These studies have indicated that the distribution of second heart field derived cells is affected in *Nos3−/−* and that Notch signaling plays a critical role in the formation of the intercalated cushion. Interestingly, the nitric oxide and Notch signaling pathways genetically interact in vivo [21]. Therefore, anomalous in coordination of these distinct cell types during arterial valve formation might be involved in BAV.

We have recently shown that the transcription factor Krox20 (also called Egr2) plays an important role in aortic valve formation [4,12,22]. Indeed, *Krox20−/−* mice develop aortic insufficiency associated with partially penetrant R-N BAVs. Similar defect is observed when Krox20 expression is inactivated in neural crest or endothelial lineages indicating that Krox20 functions in different cell types during valve development. BAV has also been found in mice deficient in *Nkx2-5*, *Hoxa1*, *Gata5*, *Gata6*, *slit*/*robo*, *Notch1*, and *Nos3* [16,23,24,25,26,27]. Recently, BAV phenotype in *Nos3−/−* has been associated to a small deviation in the distribution of distinct valvular cell types [20]. Here, we show that *Krox20* and *Nos3* genetically interact in vivo as compound heterozygous *Krox20;Nos3* mutant mice display BAV malformations whereas single heterozygous mutant mice have normal aortic valves. We further demonstrate that Krox20 regulates *Nos3* expression by direct activation of *Nos3* proximal promoters during arterial valve development. We used aortic valve interstitial cells (AVICs) in vitro model to confirm this activation. Conversely, we found normal contribution of *Krox20*-Cre labeled cells in *Nos3* mutant mice. Our study thus provides new mechanistic insights into the regulation of nitric oxide activity during the formation of the aortic valve and disease such as BAV.

## 2. Materials and Methods 

### 2.1. Mice

All animal procedures were carried out under protocols approved by a national appointed ethical committee for animal experimentation (Ministère de l’Education Nationale, de l’Enseignement Supérieur, de la Recherche et de l’innovation; APAFIS #2931-2015113016228473) and conformed to Directive 2010/63/EU of the European Parliament. Genotyping of *Nos3+/−* and *Krox20+/−* mice was performed as previously described [28,29]. Compound mutant mice were obtained by intercross *Krox20+/−* with *Nos3+/−* mice. The *Krox20^Cre^* and *Krox20^flox^* alleles and *Tie2-cre*, *Wnt1-cre*, −31/−23.5 *Krox20/LacZ,* and *Gt(ROSA)26Sor^tm9(CAG-tdTomato)Hze^ (Rosa^tdTomato^)* transgenic mice have been previously described [4]. 

### 2.2. Real-Time qRT-PCR

The OFT and aortic valve leaflets were manually dissected from E13.5 and 18.5 mutant embryos. After genotyping, samples from 5 mice of the same genotype were used and RNA isolated using NucleoSpin RNA/Protein kit (Macherey-Nagel, Düren, Germany) per manufacturer’s instructions. Reverse transcriptions were performed by using first strand cDNA synthesis kit (Roche, Basel, Switzerland) per manufacturer’s instructions. LightCycler 480 SYBR Green I Master mix (Roche, Basel, Switzerland) was used for quantitative real-time qRT-PCR analysis with a LightCycler 480 (Roche, Basel, Switzerland) following the manufacturer’s instructions. Gene-specific primers used in this study are listed in Table 1. Each experiment was performed in triplicate for each genotype. Samples were normalized to endogenous housekeeping gene (*TBP* gene). Level changes were calculated by the comparative cycle threshold (ΔΔCT) method. Normalized expression levels in the control were set to 1.0 for each gene.

### 2.3. Histological and Immunostaining

Staged mouse hearts were fixed 1 h in 4% paraformaldehyde, washed in phosphate buffered saline, and then paraffin embedded, sectioned at 8-μm, and then processed as previously described [4]. Sections were stained with hematoxylin & eosin (H & E) (Sigma-Aldrich, St. Louis, MO, USA) according to the manufacturer’s instruction. X-gal staining was performed as previously described [4]. Sections or whole-mount embryos were examined using an Axio Zoom. V16 (Zeiss, Oberkochen, Germany) was photographed with an Axiocam digital camera (Zen 2011, Zeiss).

Polyclonal anti-Nos3 antibody was purchased from Microm (Rabbit, 1:50) and used on OCT embedded and cryo-sectioned fixed tissue. The anti-Pecam (CD31, Rat, 1:100) antibody was purchased from BD-Pharmingen (BD Biosciences, San Jose, CA, USA). The Alexa fluorescent-conjugated antibody (Life Technologies, Thermo Fischer Scientific, Carlsbad, CA, USA) was used at 1:500. Images were taken with DM5000 microscope with LAS software (Leica Microsystems, Wetzlar, Germany). For each experiment, a minimum of 3 embryos of each genotype was scored. 

### 2.4. DNA-Binding Assay

For electrophoretic mobility shift assays (EMSA), the Krox20 protein was produced with the TNT (T7)-coupled in vitro transcription/translation system (Promega, Madison, WI, USA) as previously described [12]. The Probes used for KROX binding corresponded to the -513 and -136 KROX binding sites (Table 1). For EMSA, 3 or 9-μL of in vitro translated Krox20 protein were mixed in 20-μL binding reaction containing 20% glycerol, 50 mM Tris-HCl pH 7.5, 250 M NaCl, 2.5 mM EDTA, 2.5 mM DTT, and 5 mM MgCl2. The reactions were incubated on ice for 10 min before the addition of 10,000 counts/min [α-^32^P]dATP-labeled oligonucleotides. Mixtures were further incubated on ice for 30 min before being loaded on a 0.5× Tris-boric acid-EDTA buffer-4% polyacrylamide gel, and then electrophoresis was carried out at 250 V for 1.5 h at 4 °C. The gel was dried and exposed to a Kodak autoradiography film overnight at −80 °C.

### 2.5. Chromatin Immunoprecipitation (ChIP)

For ChIP experiments, hearts at stage E13.5 were collected and dissected in cold PBS. Freshly dissected OFT and left ventricles were lysed in trypsin solution and homogenized in cold PBS containing a protease inhibitor cocktail (Roche, Basel, Switzerland). Tissues were fixed in 1% formaldehyde for 15 min at RT and 40 min at 4 °C on a shaking platform. Formaldehyde cross-linking was stopped by adding Glycine to a final concentration of 0.125 M and incubated for 5 min at RT. Tissues were then lysed in lysis buffer (0.5% NP-40, 5 mM PIPES pH 8.0, 85 mM KCl) containing a protease inhibitor cocktail and then homogenized using dounce homogenizer. Nuclei lysates were collected, lysed in Nuclei lysis buffer (1% SDS, 50 mM Tris-HCl pH 8.0, 10 mM EDTA) containing a protease inhibitor cocktail and then sonicated to obtain chromatin fragments <1 kb. Chromatin was diluted 1:10 with ChIP Solution (1% Triton-X100, 0.01% SDS, 1.2 mM EDTA, 167 mM NaCl, and 16.7 mM Tris-HCl pH 8.0) containing protease inhibitor cocktail and precleared with 50% salmon sperm DNA/protein-A sepharose slurry (Sigma-Aldrich, St. Louis, MO, USA). Chromatin fragments were incubated with one of the following antibodies at 4 °C on a rotating platform: 5 µg of rabbit polyclonal anti-Histone H3 (Millipore, Burlington, MA, USA) and 10 µg of rabbit polyclonal anti-Krox20 (Covance, Princeton, NJ, USA). Magna ChIP™ Protein A Magnetic Beads (Millipore) (25 µL) were then added and incubated for 1 h at 4 °C. Immunoprecipitated pellets were washed once with a “low salt” solution (0.1% SDS, 1% Triton-X100, 2 mM EDTA, 20 mM Tris-HCl (pH 8.0) and 150 mM NaCl), once with a “high salt” solution (0.1% SDS, 1% Triton-X100, 2 mM EDTA, 20 mM Tris-HCl (pH 8.0), and 500 mM NaCl) and once with 0.25 M LiCl, 1% Nonidet P-40, 1% sodium deoxycholate, 1 mM EDTA and 10 mM Tris-HCl, pH 8.0 and twice 10 mM Tris-HCl (pH 8.0) and 1 mM EDTA. Chromatin was eluted from the beads with 250 µL 1% SDS and 0.1 M NaHCO_3_. Crosslinks were reversed for 4 h at 65 °C after addition of 20 µL of 5 M NaCl. Samples were supplemented with 20 µL of 1 M Tris-HCl (pH 7.0), 10 µL of 0.5 M EDTA, and 40 µg of proteinase K and incubated for 1 h at 45 °C. DNA was then recovered by phenol/chloroform extraction and ethanol precipitation. For total DNA samples (Input), aliquots corresponding to 1:10 dilution of the amount lysate used in the immunoprecipitation were processed along the rest of samples at the step of reversing the crosslink. DNA samples were quantified using the NanoDrop ND-1000 spectrophotometer (NanoDrop Technologies LLC, Wilmington, DE, USA). The presence of individual Krox20 consensus binding site on *Nos3* promoters was analyzed by qPCR using LightCycler 480 SYBR Green I Master mix (Roche, Basel, Switzerland) on a LightCycler480 (Roche, Basel, Switzerland) following manufacturer’s instructions. The Histone H3 antibody was used as positive control of immunoprecipitation and a set of primers for *Nos3* corresponding to a coding region without consensus binding site were used as negative control. Relative quantities of each chromatin bound fragment expression were calculated using the comparative cycle threshold (ΔΔCT) method and were normalized to the amount of input DNA (in the same amount of chromatin before immunoprecipitation, quantified with the same PCR), and to the level of *TBP* gene. Primers used in this experiment were listed in Table 1.

### 2.6. Luciferase Assay

The *Krox20* expression plasmid (CMV-*Krox20*) has been previously described [12]. The Nos3 constructs were derived from the pXP2 reporter constructs (kindly provided by Pr. Mona Nemer) [24]. The pXP2-Nos3-1522 and pXP2-Nos3-265 reporter constructs contain a segment extending from the bp +1 to −1522, and bp +1 to −265 of the *Nos3* proximal promoter cloned upstream the *luc* gene in the pXP2. To generate the mutated *Krox20* reporter constructs, one consensus Krox20 binding site in the *Nos3* promoter (bp +1 to −1522) was changed by PCR (K2; GTGTGGGAC to mutated K2; GTGagtcAC). Cos7 cells were transiently co-transfected using the Promofectin (Promocell, Heidelberg, Germany) according to the manufacturer**’**s instructions with 200 ng of indicated constructs and 10 ng of control pXP22 vector and various amounts (0 ng, 50 ng, 150 ng, and 300 ng) of CMV-*Krox20* expression vector or of CMV-control. Then, 24 h after transfection, cells were lysed, and luciferase activity was measured using the Dual-Luciferase^®^ Reporter Assay System (Promega, Madison, WI, USA). All the transfection experiments were done in triplicate and repeated at least three times. Luciferase activities were read using a GloMax^®^-Multi Microplate Multimode Reader and were normalized to *Renilla* luciferase to compensate for variations in transfection efficiency. Results are presented as fold activation of the relative luciferase activities over the CMV control.

### 2.7. Rat Aortic Valve Interstitial Cell Culture and Transfection

Rat aortic valve interstitial cells (AVICs) were collected from aortic valve of 10-week-old Sprague Dawley females. AVICs were isolated as previously described by Gould et al., 2010 [30]. Briefly, valves leaflets were isolated and submitted to collagenase type II digestion. To isolate AVIC, leaflets were cut into small pieces and placed into collagenase solution during 8 h at 37 °C. After isolation, AVICs were cultured in DMEM-GlutamaX (Invitrogen, Thermo Fischer Scientific, Carlsbad, CA, USA) supplemented with 10% FCS (Invitrogen). AVICs were used between passage 3 and 7 for this study. AVIC were seeded into 6-well plates at 200,000 cells of density and 500 ng of CMV-Krox20 or CMV-GFP were transfected using Promofectin (Promocell, Heidelberg, Germany). Cells were lysed 24 h, 48 h, or 72 h after transfection, and RNA was isolated using NucleoSpin RNA/Protein kit (Macherey-Nagel, Düren, Germany).

## 3. Results

### 3.1. Expression of Genes Associated with BAVs

**Figure 1:**
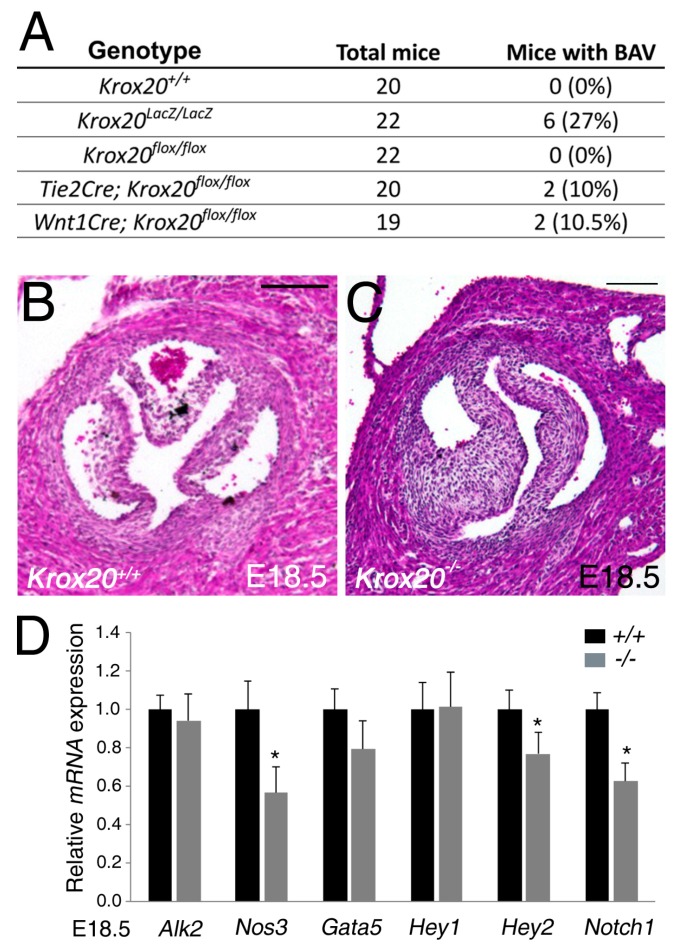
Modulation of gene expression in *Krox20−/−* embryos. (**A**) Table depicting penetrance of bicuspid aortic valve (BAV) in *Krox20−/−*, *Tie2-cre;Krox20f/f*, and *Wnt1-cre;Krox20f/f* mice. (**B**,**C**) Cross-sectional H&E images through the aortic valve of *Krox20+/+* (**B**) and *Krox20−/−* (**C**) littermate embryos. At E18.5, left and right-coronary leaflets are observed in BAV of *Krox20−/−* (**C**) embryos. (**D**) Real-time qPCR analyses were performed from isolated aortic valve of *Krox20+/+* (*n* = 5) and *Krox20−/−* (*n* = 5) embryos at E18.5. qPCR showing normal levels of *Alk2*, *Gata5*, and *Hey1*, and altered expression of *Nos3*, *Notch1*, and *Hey2* in the aortic valve of *Krox20−/−* embryos at E18.5. qRT-PCR experiments were performed in triplicate and expressed as mean ±SEM (* *p* < 0.05 using Mann–Whitney test). Scale bars: 100 μm.

We have previously reported that Krox20 is expressed in valvular cell populations contributing to arterial valve development including endothelial and neural crest derivatives [4,12]. Consistently, *Krox20−/−* mice are predisposed to develop a BAV (27%) (Figure 1A–C). Interestingly, conditional deletion of *Krox20* in the endothelial-, or neural crest cell-lineage led to BAV phenotype indicating that Krox20 is required in both lineages (Figure 1A). Several studies have shown that defective function of *Notch1*, *Gata5*, *Alk2*, and *Nos3* leads to BAV development [16,23,24,25]. To determine if the transcription factor Krox20 is playing a role in the activation of these genes, we analyzed their transcriptional levels in *Krox20−/−*. Expression of *Alk2* and *Gata5* are unchanged in *Krox20−/−* aortic valves (Figure 1D). However, a significant reduction of the mRNA levels of *Nos3* is observed in *Krox20* mutant embryos at E18.5 (Figure 1D). Interestingly, expression of *Notch1*, which is regulated by Nos3 signaling in aortic valve disease [21], is also reduced in *Krox20−/−* embryos. We also examined expression of Notch1 target genes, *Hey1*, *Hey2*. While mRNA levels of *Hey1* are unchanged in *Krox20* mutant (Figure 1D), we found a significant reduction of *Hey2* expression in the aortic valve from *Krox20−/−* compared to the littermates (Figure 1D). These findings indicate a down-regulation of genes previously associated with aortic valve disease and BAV in *Krox20−/−* mice.

### 3.2. Krox20 Interacts *In Vivo* with Nos3

**Figure 2:**
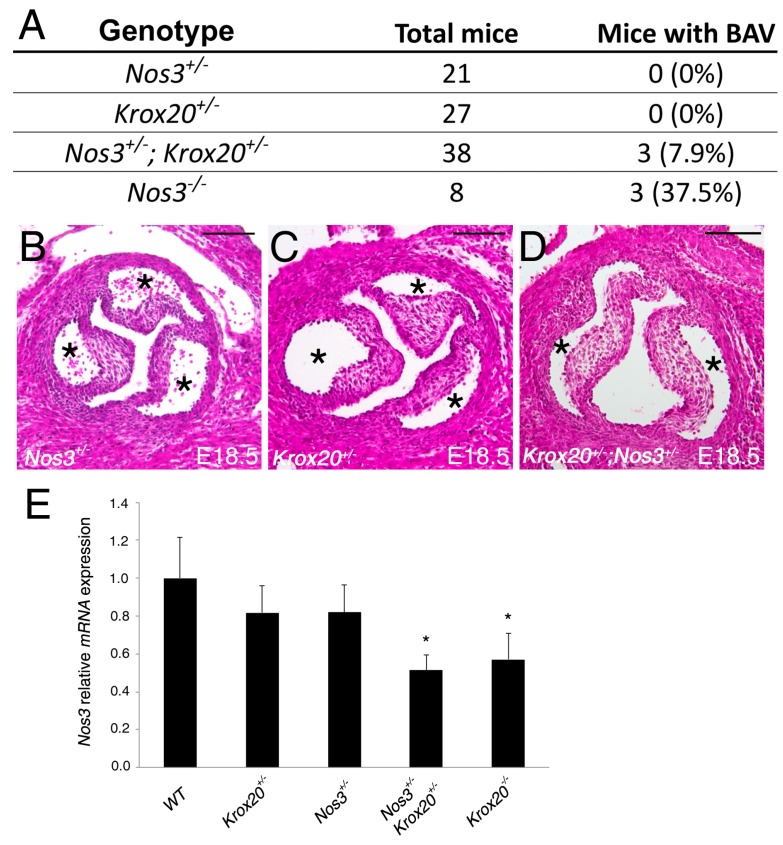
Abnormal aortic valve morphology in *Krox20+/−;Nos3+/−* mice. (**A**) Table depicting penetrance of bicuspid aortic valve (BAV) in *Nos3+/−*, *Krox20+/−*, *Nos3+/−*;*Krox20+/−* and *Nos3−/−* mice. (**B**–**D**) H&E images showing representative *Nos3+/−* and *Krox20+/−* with tri-leaflets aortic valve, and an example of *Nos3+/−*;*Krox20+/−* aortic valve with 2 leaflets. Aortic valve leaflets of *Nos3+/−*;*Krox20+/−* mice appear equal in size. (**E**) Real-time qPCR demonstrates a reduction of *Nos3* at a transcriptional level in *Nos3+/−*;*Krox20+/−* and *Krox20−/−* compared to wild-type embryos (*n* = 5 for each genotype). qPCR experiments were performed in triplicate and expressed as mean ± SEM (* *p* < 0.05 using Mann–Whitney test). Scale bars: 100 μm.

Since *Nos3* is required for normal aortic valve development, and is associated with aortic valve disease including BAV, specifically in the fusion of the right-coronary and non-coronary leaflets, we examined if Krox20 and Nos3 signaling pathways exhibited an in vivo genetic interaction in aortic valve development. As previously shown [16], at E18.5, *Nos3−/−* embryos (*n* = 8) are observed at the expected mendelian ratio with a partially penetrance of BAV (28%), whereas all *Nos3+/−* embryos (*n* = 21) have normal aortic valve (Figure 2A,B)*. Krox20* heterozygous embryos are observed at the expected mendelian ratio with no evidence of BAV (Figure 2A,C). We bred *Krox20+/−* mice with *Nos3+/−* or *Nos3−/−* mice and found low incidence of BAV in compound *Krox20+/−;Nos3+/−* embryos (8%; Figure 2A,D), suggesting a genetic interaction between these two genes. To test if transcriptional levels of *Nos3* are affected in this context, we analyzed the *Nos3* mRNA levels in single or compound mutants. Interestingly, significant decrease of *Nos3* mRNA is observed in *Krox20+/−;Nos3+/−* mice (Figure 2E). We found a similar reduction of *Nos3* mRNA in *Krox20−/−*, suggesting that Krox20 controls *Nos3* mRNA expression.

**Figure 3:**
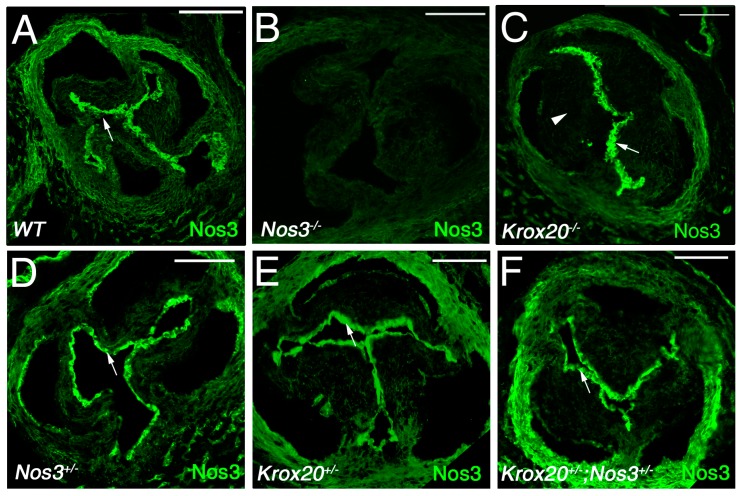
Endothelial nitric oxide synthase (Nos3) expression is altered in Krox20 deficient mice. (**A**–**F**) Immunohistochemistry showing Nos3 protein (green) in the aortic valve of wild-type (WT, **A**), *Nos3−/−* (**B**), *Krox20−/−* (**C**), *Nos3+/−* (**D**), *Krox20+/−* (**E**), and *Nos3+/−*; *Krox20+/−* (**F**) embryos at E18.5. Immunohistochemistry showing abundant expression of Nos3 in the valve endothelial cells (arrows). Note the reduction of Nos3 expression in the mesenchyme of *Krox20−/−* (**C**) aortic valve leaflets (arrowhead; compared **C** with **A**). Scale bars: 100 μm.

Since *Nos3* mRNA level is downregulated in *Krox20−/−* mice, we examined wild-type, single, and compound mutant hearts to determine whether there is a correlation with Nos3 protein expression. Therefore, we performed immunohistochemistry with anti-Nos3 and anti-Pecam on compound mutant hearts. Consistent with previous observation [11], we found Nos3 expression in the endothelial and mesenchyme of wild-type aortic valve leaflets at E18.5 (Figure 3A). As expected, no expression is detected in *Nos3−/−* embryos (Figure 3B). Although we did not observe a difference of Nos3 expression in the valvular endothelium in *Krox20−/−* mice, its expression is reduced in the mesenchyme of the aortic valve leaflets where Krox20 is normally expressed (Figure 3C). The expression of Nos3 is unaffected in *Nos3+/−* or *Krox20+/−* mice. Anti-Pecam immunostaining shows that integrity of the endothelium is maintained in all genotypes analyzed (Figure S1). Altogether, these data indicate that mesenchymal Nos3 expression is dependent on Krox20. To assess whether the Krox20-dependent Nos3-expressing mesenchyme cells are derived from the endothelial-, or neural crest cell-lineage, we performed qPCR on aortic valves from *Tie2-cre;Krox20f/f* and *Wnt1-cre;Krox20f/f* mice (Figure S2). Transcriptional expression of *Nos3* is reduced in both conditional mutant mice, suggesting that Nos3 is impaired in mesenchyme cells derived from both lineages.

### 3.3. Krox20 Activates Nos3 Promoter

**Figure 4:**
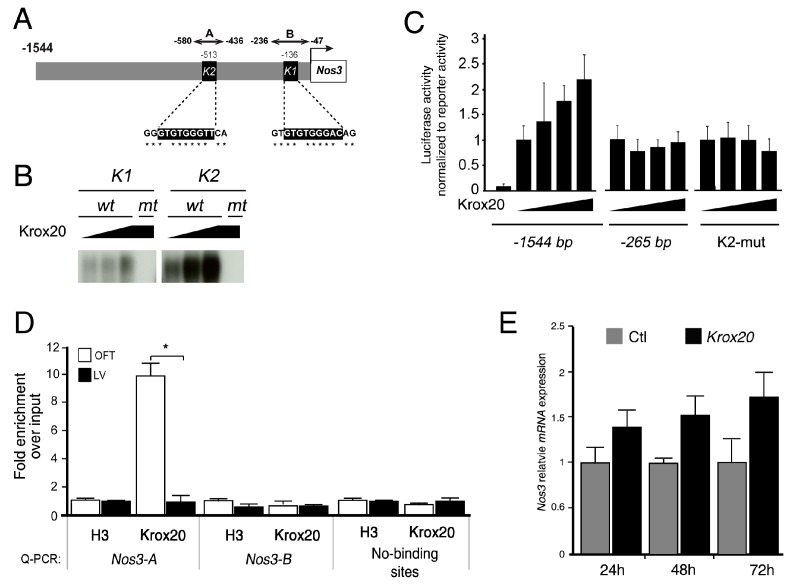
Krox20 promotes the transcriptional activity of the *Nos3* proximal promoter. (**A**) Schematic representation of the 1.5-kb *Nos3* proximal promoters. The putative Krox20 binding sites located around −513 bp and −136 bp on *Nos3* promoters are represented by black squares. Asterisk (*) indicates conservation of Krox20-binding sites with human and rat sequences. Numbers indicate position of the sequences from the ATG. (**B**) EMSA showing binding of Krox20 to the wild-type K1 (GTGTGGGAC) and K2 (GTGTGGGTT) motifs. Mutation of the K1 and K2 motif impairs Krox20 binding. (**C**) Relative luciferase activity in Cos7 cells transiently co-transfected with reporter constructs containing a 1544 bp and 265 bp regions of *Nos3* promoter cloned upstream of the luciferase gene, 10 ng of control pGL4.74(*hRluc*/TK) vector and 50, 150, and 300 ng of CMV-Krox20 or CMV control expressing vectors. Transfection of CMV-*Krox20* has a trans-activating effect on −1544 bp reporter. Mutation of the K2 motif abolishes the trans-activation of Krox20 on the −1544 bp reporter. Data is represented as a fold change in luciferase activity normalized to *Renilla*. (**D**) qPCR showing significant enrichment of DNA/Krox20 complexes on Krox20-binding sites within the *Nos3* (regions A) proximal promoters following chromatin immunoprecipitation using dissected outflow tract (OFT) and left ventricle (LV) from E13.5 hearts. Anti-Histone H3 was used as positive control of immunoprecipitation. Primers used for qPCR correspond to *Nos3* proximal promoter region (*Nos3-A*, *Nos3-B*) as indicated in (**A**). Primers in region without Krox20 binding sites (no binding site) were used as a negative control. Relative quantities of each chromatin bound fragment were normalized relative to the amount of input DNA. Note significant enrichment in region A within the *Nos3* promoters. (**E**) *Nos3* transcriptional level was quantified by qRT-PCR in rat AVICs, 24 h, 48 h, and 72 h after *Krox20* transfection. Experiments were performed in triplicate and expressed as means ± SEM.

Laforest and colleagues have identified conserved GATA binding sites in the murine −1.5-kb promoter of *Nos3* and showed that Gata5 enhanced *Nos3* promoter activity through these binding sites [24]. Based on these data, we examined the *Nos3* promoter for conserved Krox20 binding sites. Bioinformatics analysis identified two evolutionary conserved Krox20-binding sites (K1 and K2) [31] in the *Nos3* proximal promoter (Figure 4A). EMSA experiments showed that Krox20 is able to bind to these binding sites with a higher affinity for the K2 site (Figure 4B). Additionally, we carried out luciferase assays to show that overexpression of Krox20 in Cos7 cells increased the transcriptional activity of the −1500 bp region up to 2.5-fold but not the −265-bp *Nos3* promoter (Figure 4C). Importance of K2 site is confirmed by luciferase assay, as mutation of this specific site abolished the activation of the −1500 bp promoter by Krox20. To confirm binding of Krox20, we performed chromatin immunoprecipitation (ChIP) experiments on freshly dissected OFT and ventricle tissues at E13.5. The amount of immunoprecipitated DNA relative to the input chromatin is determined by quantitative PCR using primers flanking Krox20-binding sites in the *Nos3* (A and B regions) proximal promoters (Figure 4). Consistent with the high affinity of Krox20 for the K2 site and the transactivation results, ChIP qPCR demonstrated enrichment of binding only within the A region of *Nos3* in the OFT extracts (Figure 4D). Furthermore, transfection of rat AVIC with *Krox20* is sufficient to activate Nos3 expression (Figure 4E). Together, these results identified Krox20 as an activator of *Nos3* promoter and suggest that reduction of *Nos3* expression may be one of the leading causes of BAVs observed in *Krox20* mutant mice.

### 3.4. Krox20 Is Not Affected in Nos3 Mutant Mice

**Figure 5:**
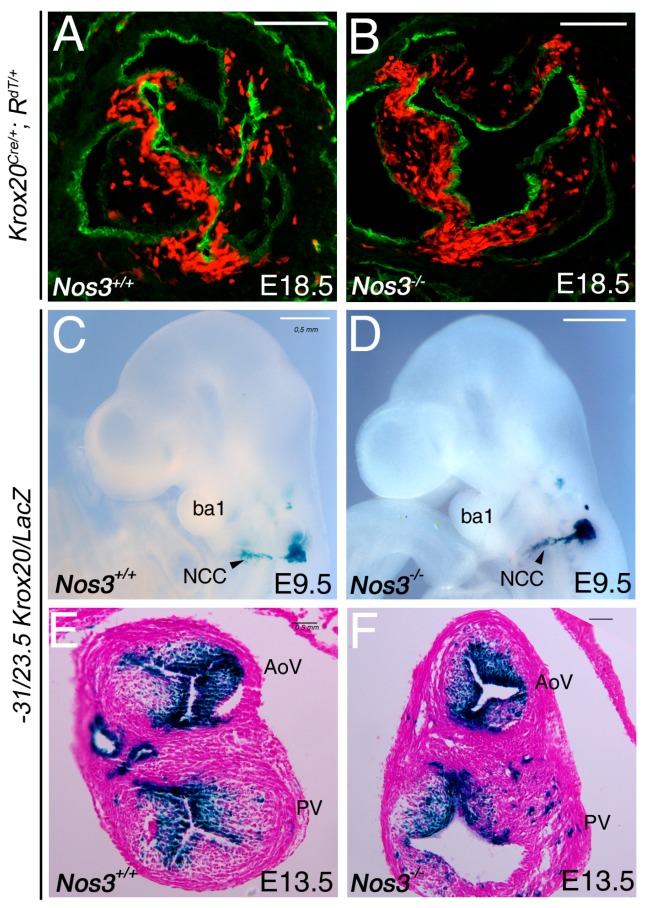
Absence of *Nos3* does not affect Krox20 contribution. (**A**,**B**) Embryos were harvested from *Krox20^Cre/+^;Rosa^tdTomato^* mice at E18.5 and immunohistochemistry is performed to detect Tomato expression (red) as an indicator of recombination, while Pecam (green) identifies endothelial cells in the aortic valve. No major difference is observed between *Nos3−/−* and control littermate embryos. (**C**–**F**) X-gal staining was performed on −31/−23.5 *Krox20/LacZ* transgenic mice to follow the *Krox20*-expressing cells at E9.5 (**C**,**D**), and E13.5 (**E,F**) stages. (**C**,**D**) At E9.5, β-galactosidase (β-gal) activity is detected in migratory neural crest cells. No defect is observed in *Nos3−/−* compared with control embryos (compared **D** with **C**). (**E**,**F**) Transverse section through the outflow tract cushions at E13.5 showing β-gal-positive cells in the arterial valve leaflets. AoV, aortic valve; ba1, branchial arch 1; PV, pulmonary valve; NCC, neural crest cells. Scale bars: 100 μm (**A**,**B**); 50 μm (**C**–**F**).

To determine whether nitric oxide is regulating *Krox20*, we examined the *Krox20*-labeled cells in *Nos3* mutant mice. Thus, we used *Krox20^Cre^* and *Rosa-tdTomato* mouse lines to perform lineage tracing of *Krox20*-labeled cells in wild-type and *Nos3-*null mutant backgrounds. Immunostaining detected Tomato expression (red), as an indicator of recombination, and Pecam (green) as an endothelial cell marker (Figure 5A). As previously observed, *Krox20*-lineage contributes to mesenchymal cells of the aortic valve leaflets (Figure 5A). At E18.5, the number of recombined cells is comparable between *Nos3−/−* and wild-type embryos (Figure 5A,B). To further follow the migration of the neural crest cells that contribute to the formation of the arterial valves, we used the −31/−23.5 *Krox20/LacZ* transgene [4]. At E9.5, we found no reduction of X-gal-positive cells in *Nos3−/−* compared to wild-type littermate embryos (Figure 5C,D). At E13.5, X-gal staining confirmed the earlier observation (Figure 5E,F). Together, these results indicate that absence of *Nos3* did not affect the contribution of Krox20 during valve development.

## 4. Discussion

Here, we report BAV phenotype in compound heterozygous *Krox20;Nos3* mice, and show a direct activation of *Nos3* expression by Krox20, demonstrating a genetic link between these two genes already known to be implicated in aortic valve disease including BAV [4,17]. We have previously shown that *Krox20* is expressed in mesenchymal cells of the aortic valve leaflets, and that its function is required in both endothelial and neural crest derivatives to form normal aortic valves [4,12,22]. Aortic valve endothelial and interstitial cells play important roles in the development and remodeling of the aortic valve, as their dysfunction has been associated to BAV. Mice lacking functional Nos3 demonstrated a partially penetrance of BAV phenotype [3,16]. This observation is supported by a study showing that patients with BAV display decreased levels of *Nos3* [17]. While, molecular basis of BAV in *Nos3−/−* mice is still unknown, it has been showed that EndMT is impaired in the AVC cushions of *Nos3−/−* embryos at E12.5 [32]. However, its function in the OFT cushions has not yet been reported. Interestingly, a recent study uncovered a novel mechanism by which nitric oxide modulates gene expression in neighboring cells, including expression of *Hey1*, a downstream mediator of Notch1 signaling [21]. This study also revealed an in vivo genetic interaction between *Nos3* and *Notch1*, which has already been associated with BAVs in humans and mice [23]. Interestingly, expression of *Notch1* and its downstream effector *Hey2* are reduced in *Krox20−/−* mice, indicating that absence of *Krox20* affects both nitric oxide and consequently Notch signaling during aortic valve formation. While we have no evidence for a direct regulation of Notch1 expression, our data supports a direct activation of *Nos3* by Krox20. First, our findings revealed an in vivo genetic interaction between *Krox20* and *Nos3* mutant alleles, as BAV phenotype is detected in compound heterozygous but not in single heterozygous mutant mice. Second, the absence of Krox20 activity affects transcriptional levels of *Nos3* at E18.5 when BAVs are observed. Third, the 1.5-kb proximal promoter of *Nos3*, contains multiple conserved sequences that constitute putative sites for Krox20 binding. EMSA shows that Krox20 can bind K2 site with better affinity than K1 site, a result confirmed by in vivo quantitative ChIP analysis notably in the OFT, where Krox20 is strongly bound to region A of the *Nos3* promoter. Fourth, transient transfection experiments displayed that overexpression of Krox20 has transactivation effects on reporter constructs containing −1.5-kb 5′ to the *Nos3* promoter and that Nos3 expression is increased in AVIC after *Krox20* transfection. Consistently, when a shorter version of *Nos3* promoter is used, or when K2 motifs are mutated, transactivation is significantly decreased for *Nos3* promoter. Finally, *Nos3* expression is upregulated in AVICs after Krox20 transfection. These findings identify *Nos3* as a Krox20 target and suggest that reduction in Nos3 activation may be a contributing mechanism to BAV in *Krox20* mutant mice. A previous study has reported that Gata5 regulates *Nos3* expression during aortic valve formation [24]. Interestingly, GATA binding sites are located next to those of Krox20. This observation suggests that Krox20 may interact with other factors to activate *Nos3* and promote nitric oxide activity during the valvulogenesis. It has been shown that BAV is highly heritable, but with reduced penetrance and variable expressivity [33]. The high heterogeneity of BAV is probably the result of a combination of genetic, functional, and hemodynamic factors acting as modulators on the phenotype [34,35,36]. Therefore, the low percentage of BAV observed in the compound *Krox20+/−;Nos3+/−* mice suggests that the combination of the two alleles would favor the appearance of this defect. It would be interesting to study the influence of hemodynamic forces on the penetrance of BAV defect.

Because valves are constantly exposed to hemodynamic forces, it is critical to understand how shear stress is associated to valve remodeling [37]. The blood flow mediates nitric oxide production through influencing Nos3 expression and activity [38]. Nos3 is abundantly expressed in the valve endothelial cells but nitric oxide is also known to regulate VIC phenotypic expression in the aortic valve [39]. Moreover, porcine AVICs treated with agonist or antagonist of Nos3 demonstrated that nitric oxide activity impact AVIC phenotype [21,40]. Here, we show that Nos3 is expressed in the mesenchymal cells (or VICs) during development of the aortic valves, and that transient transfection of Krox20 in rat AVICs activates *Nos3* expression (Figure 4E). This observation is consistent with another study which has reported Nos3 expression in the mesenchyme of the aortic valve [11]. The decrease of *Nos3* expression in *Krox20* mutant mice may impact mesenchymal differentiation and the remodeling of the aortic valve. We recently observed that Krox20 expression is modulated in AVICs in response to different shear stress forces (unpublished data). These observations suggest that Krox20 may participate to the regulation of nitric oxide during aortic valve leaflet development. A potent activator of the AVICs is the transforming growth factor-β1 (TGF-β1) [41]. TGF-β1, as well as other inflammatory cytokines, regulates the activation of nitric oxide in normal and pathological valves. Further investigation is required to determine if Krox20 interacts with other pathways to regulate the nitric oxide in response to normal and pathological blood flow patterns at the surface of the aortic valve leaflets.

In *Krox20* mutant mice, aortic valve leaflets are misshaped due to an excess of neural crest derivatives [4]. This enlargement blocks normal valvulogenesis and results in BAV in 30% of *Krox20−/−* mice. The BAV in *Krox20−/−* mice results from fusion of either the right or left-coronary and non-coronary leaflets (R-N). This fusion pattern is consistent with the *Nos3* deficient mouse model of BAV where endothelial–mesenchymal signaling is impaired [3]. Our previous study indicates that Krox20 is required within the endothelial-derived cells for proper aortic valve remodeling [12]. Krox20 is also required in neural crest-derived cells as demonstrated by the BAV observed in *Wnt1-cre;Krox20f/f* mice (Figure 1A) [4]. However, the bicuspid phenotype obtains in *Tie2-cre;Krox20f/f* mice is different from those observed in *Krox20−/−* or *Wnt1-cre;Krox20f/f* mice as the BAV is formed by equal-sized leaflets. These results suggest that Krox20 plays multiple roles during the development and remodeling of the aortic valves. 

## 5. Conclusions

In conclusion, the data presented here are consistent with a crucial role for Krox20 in aortic valve development and suggest that Krox20 may be related to aortic valve disease in human. Observations that Krox20 regulates nitric oxide activity through activation of *Nos3* proximal promoter in vivo and in vitro suggest that interaction between different pathways may be implicated in aortic valve disease such as BAV. Future studies aimed at elucidating the downstream targets of Krox20 in valve cells will contribute to understanding molecular mechanics in aortic valve development as well as BAV.

## Figures and Tables

**Table 1:** Primers qPCR, ChIP and EMSA.

Name	Sequence
*Alk2*	Forward 5′- GAAGATGACGTGTAAGACCCC - 3′
	Reverse 5′- ATAAGGCCAACTTCCAGGTG - 3′
*Gata5*	Forward 5′- CTATCTATGCAATGCCTGCG - 3′
	Reverse 5′- CAGTATGGCAGTTGGAGCAG - 3′
*NOS3*	Forward 5′- CCTAGAGCACGAGGCACTG - 3′
	Reverse 5′- GTTGTACGGGCCTGACATTT - 3′
*Notch1*	Forward 5′- CAAGAGGCTTGAGATGCTCC - 3′
	Reverse 5′- AAGGATTGGAGTCCTGGCAT - 3′
*Hey1*	Forward 5′- ATGCTCAGATAACGGGCAAC - 3′
	Reverse 5′- CACCTGAAAATGCTGCACAC - 3′
*Hey2*	Forward 5′- TGAAAAACAAGGATCTGCCA - 3′
	Reverse 5′- AAGAGCATGGGCATCAAAGT - 3′
*TBP*	Forward 5′- CCCCACAACTCTTCCATTCT - 3′
	Reverse 5′- GCAGGAGTGATAGGGGTCAT - 3′
*NOS3 (rat)*	Forward 5′- GCAGTACCAGCCAGGGGA -3′
	Reverse 5′- AGGGCCACCAGGGCTGCCT -3′
*TBP (rat)*	Forward 5′- ACCCCACAACTCTTCCATTC -3′
	Reverse 5′- GGGTCATAGGAGTCATTGGTG -3′
*NOS – postATG*	Forward 5′- CTGGGTTTAGGGCTGTGC - 3′
	Reverse 5′- CTGTGGTCTGGTGCTGGTC - 3′
*NOS3-K1*	Forward 5′- CTTCCTGCTCCTTTGTGTCC - 3′
	Reverse 5′- TCCTATCTCAGAGTCCTTTGG - 3′
*NOS3-K2*	Forward 5′- TGGGTTCCCACTTATCAGCTC - 3′
	Reverse 5′- CTTTTCCTTAGGAAGCAGGGA - 3′
**EMSA**	
*K1*	Wild-type 5′- tGAGTCATGGGGTGTGGGTTCAGGAAATTGAGAT - 3′
	Mutated 5′- tGAGTCATGGGGTGagtcTTCAGGAAATTGAGAT - 3′
*K2*	Wild-type 5′- tCCTGTCCCATTGTGTGTGGGACAGGGGCGGGGCGAA – 3′
	Mutated 5′- tCCTGTCCCATTGTGTGagtcACAGGGGCGGGGCGAA – 3′

## Supplementary Materials

The following are available online at https://www.mdpi.com/2308-3425/6/4/39/s1, Figure S1: Pecam expression showing integrity of the endothelium in all genotypes, Figure S2: *Nos3* expression in *Tie2-cre;Krox20f/f* and *Wnt1-cre;Krox20f/f* embryos at E18.5.
