# Supplementary material for: Krox20 Regulates Endothelial Nitric Oxide Signaling in Aortic Valve Development and Disease
Source: J Cardiovasc Dev Dis. 2019 Nov 2;6(4):39. doi: 10.3390/jcdd6040039 (PMC6955692; doi:10.3390/jcdd6040039)
Supplement: Supplementary file 1 [file jcdd-06-00039-s001.pdf]

Supplementary data

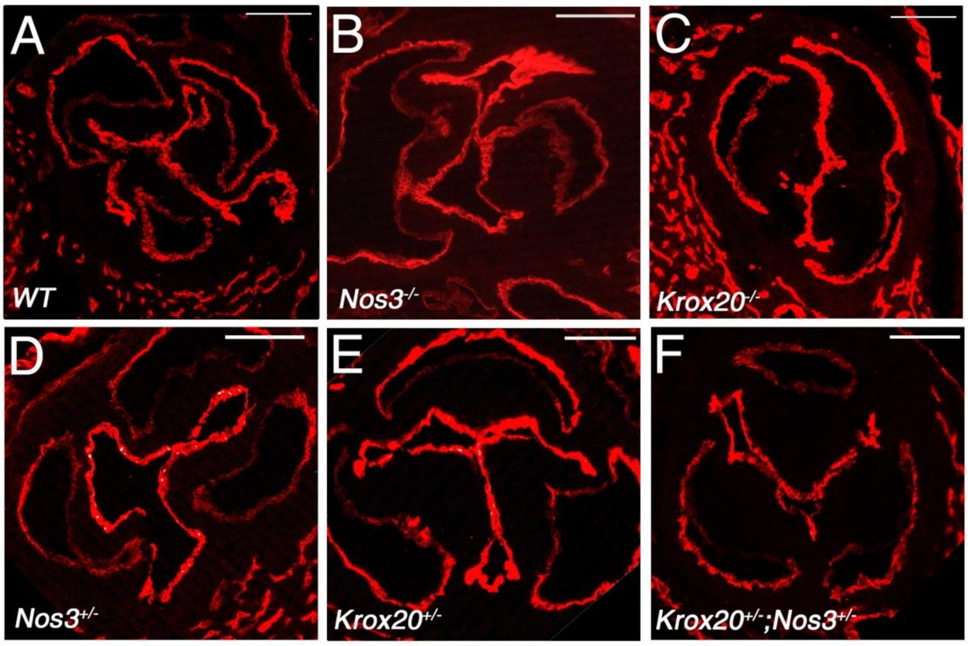

Figure S1: Pecam expression showing integrity of the endothelium in all genotypes. (**A**-**F**) Immunohistochemistry showing Pecam protein (red) in the aortic valve of wild-type (WT, **A**), *Nos3-/-* (**B**), *Krox20-/-* (**C**), *Nos3+/-* (**D**), *Krox20+/-* (**E**) and *Nos3+/-;Krox20+/-* (**F**) embryos at E18.5. Scale bars: 100 μm.

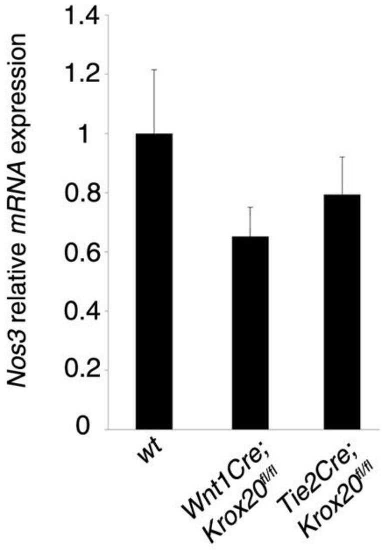

Figure S2: *Nos3* expression in *Tie2-cre;Krox20f/f* and *Wnt1-cre;Krox20f/f* embryos at E18.5. Real-time qPCR demonstrates a mild reduction of *Nos3* at a transcriptional level in *Tie2-cre;Krox20f/f* and *Wnt1-cre;Krox20f/f* compared to wild-type embryos (n=5 for each genotype). qPCR experiments were performed in triplicate and expressed as mean ±SEM.
